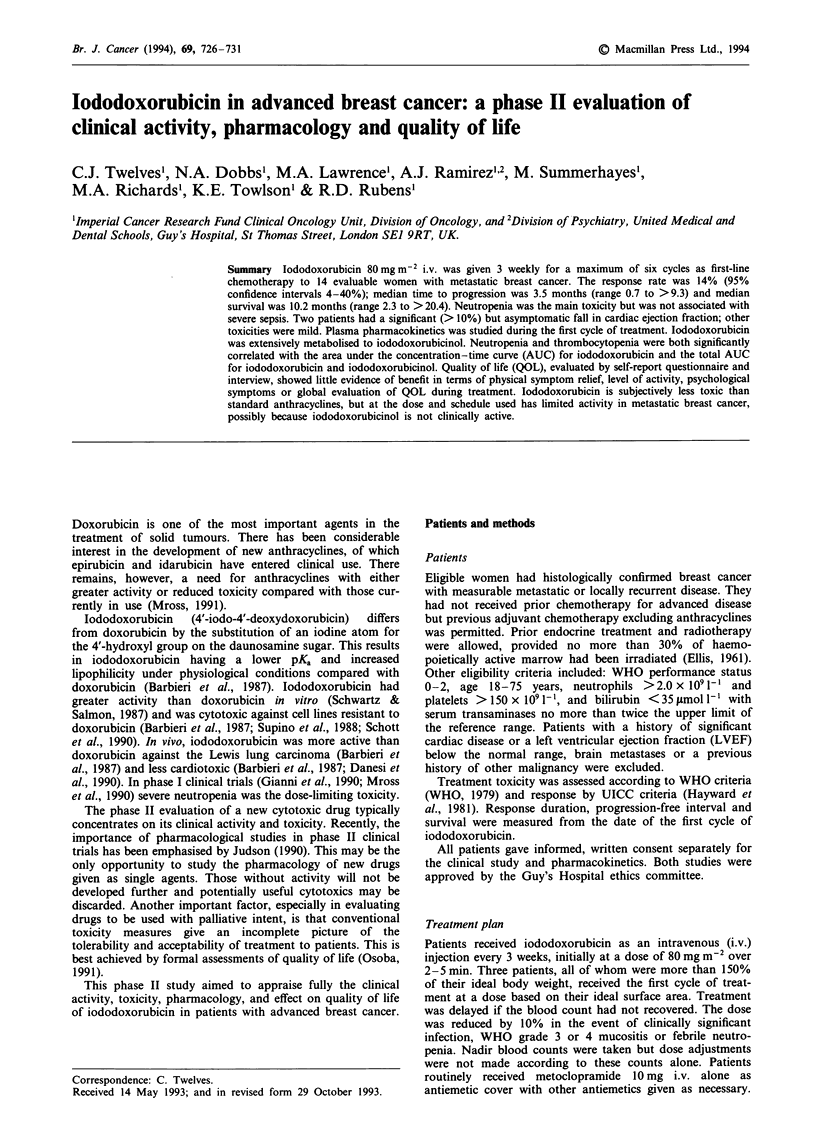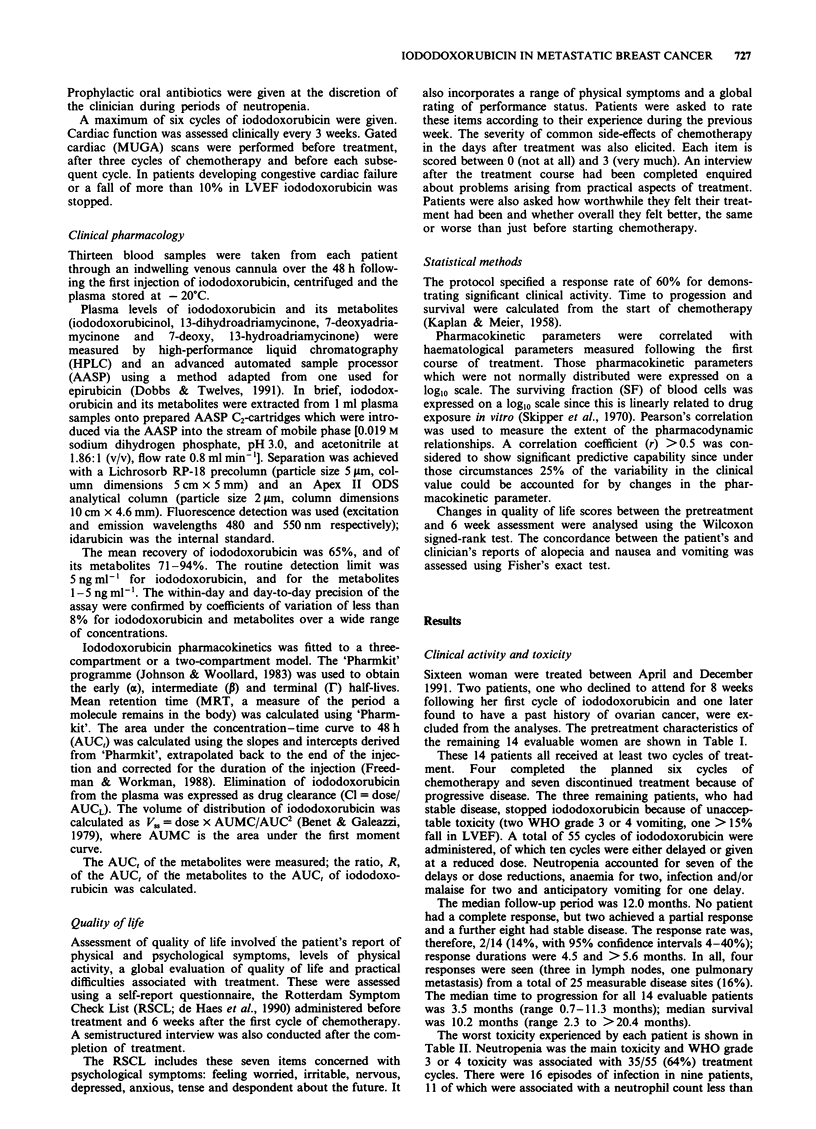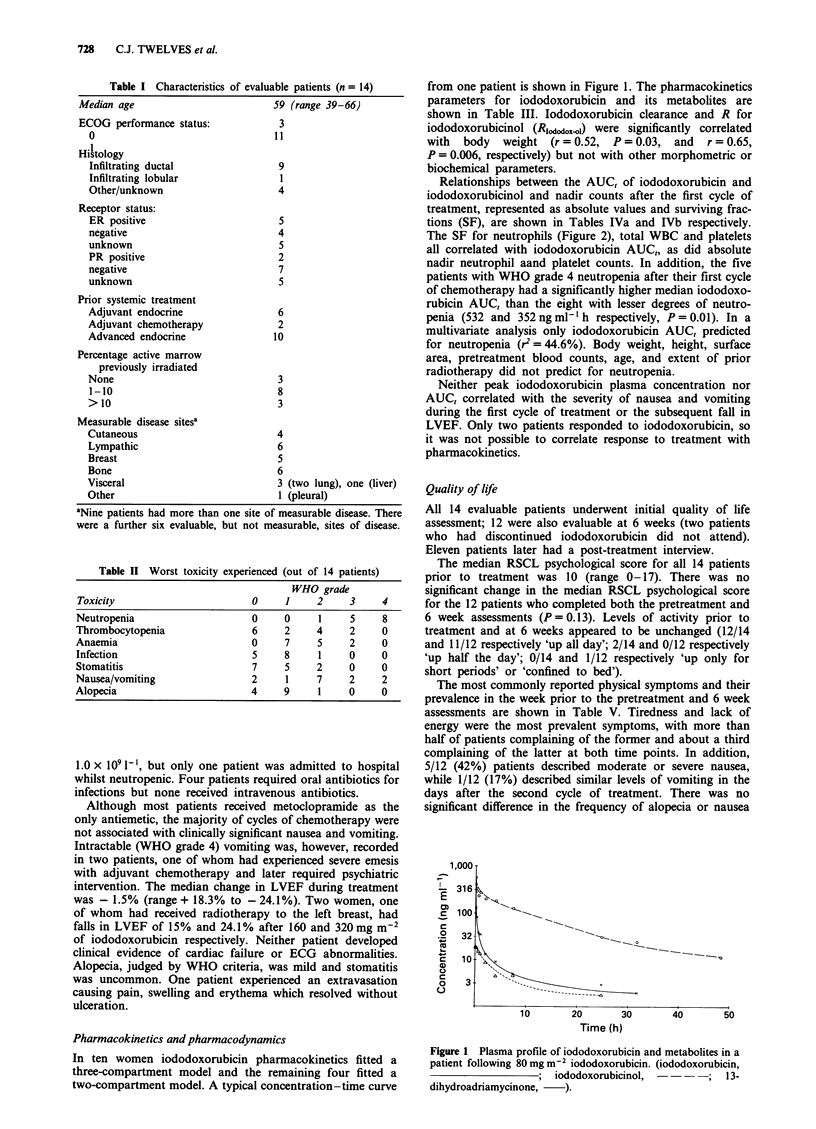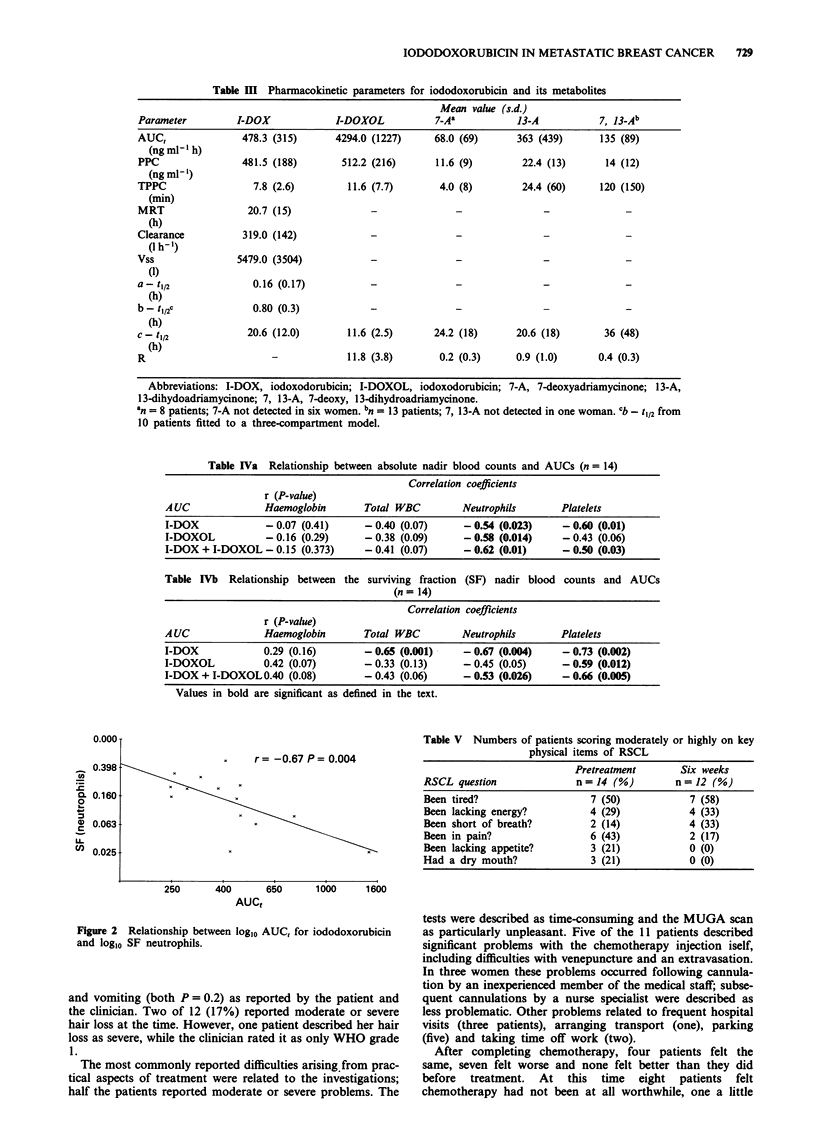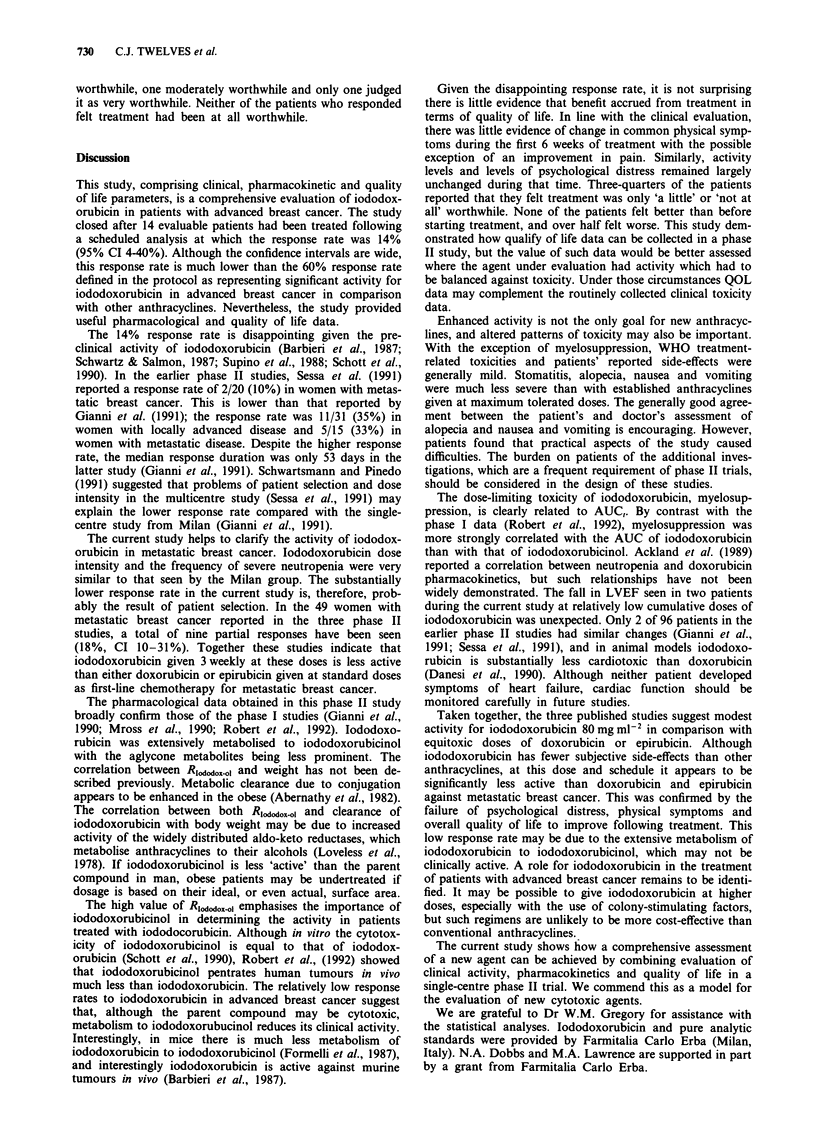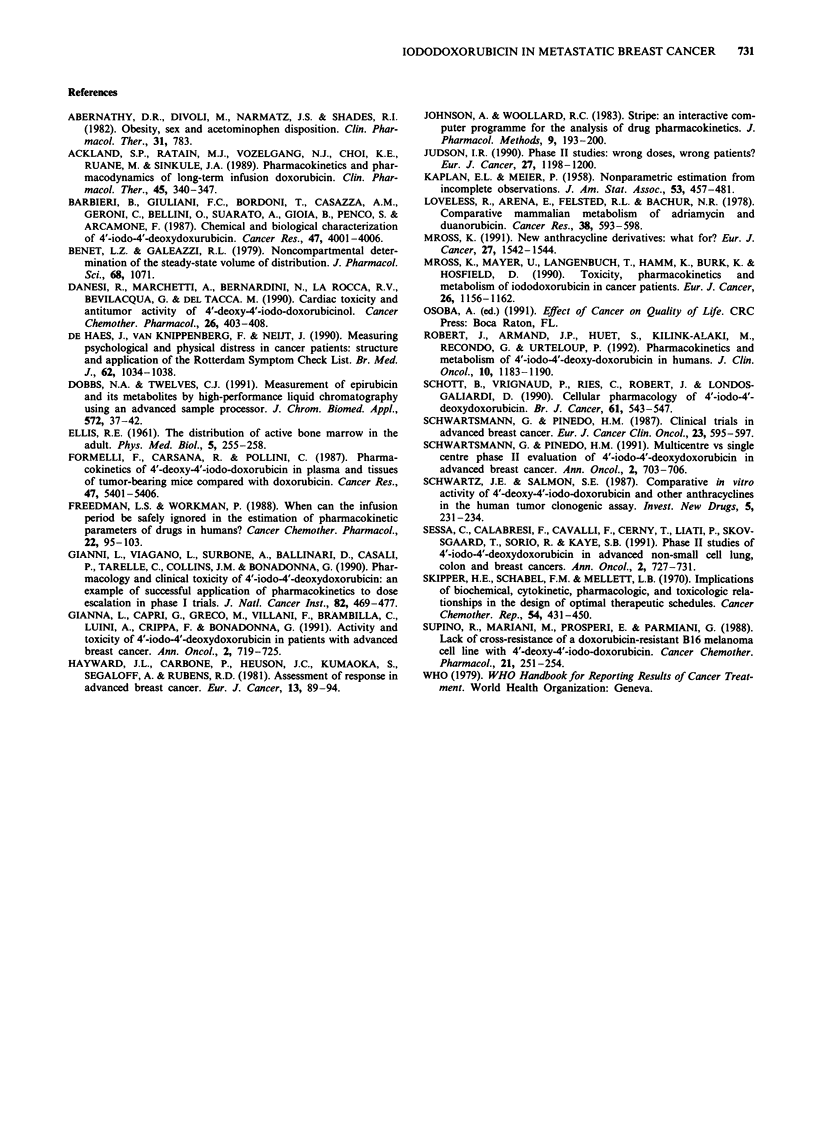# Iododoxorubicin in advanced breast cancer: a phase II evaluation of clinical activity, pharmacology and quality of life.

**DOI:** 10.1038/bjc.1994.137

**Published:** 1994-04

**Authors:** C. J. Twelves, N. A. Dobbs, M. A. Lawrence, A. J. Ramirez, M. Summerhayes, M. A. Richards, K. E. Towlson, R. D. Rubens

**Affiliations:** Imperial Cancer Research Fund Clinical Oncology Unit, United Medical School, Guy's Hospital, London, UK.

## Abstract

Iododoxorubicin 80 mg m-2 i.v. was given 3 weekly for a maximum of six cycles as first-line chemotherapy to 14 evaluable women with metastatic breast cancer. The response rate was 14% (95% confidence intervals 4-40%); median time to progression was 3.5 months (range 0.7 to > 9.3) and median survival was 10.2 months (range 2.3 to > 20.4). Neutropenia was the main toxicity but was not associated with severe sepsis. Two patients had a significant (> 10%) but asymptomatic fall in cardiac ejection fraction; other toxicities were mild. Plasma pharmacokinetics was studied during the first cycle of treatment. Iododoxorubicin was extensively metabolised to iododoxorubicinol. Neutropenia and thrombocytopenia were both significantly correlated with the area under the concentration-time curve (AUC) for iododoxorubicin and the total AUC for iododoxorubicin and iododoxorubicinol. Quality of life (QOL), evaluated by self-report questionnaire and interview, showed little evidence of benefit in terms of physical symptom relief, level of activity, psychological symptoms or global evaluation of QOL during treatment. Iododoxorubicin is subjectively less toxic than standard anthracyclines, but at the dose and schedule used has limited activity in metastatic breast cancer, possibly because iododoxorubicinol is not clinically active.


Br. J. Cancer (1994), 69, 726-731 © Macmillan Press Ltd., 1994

### Iododoxorubicin in advanced breast cancer: a phase II evaluation of clinical activity, pharmacology and quality of life

C.J. Twelves[1], N.A. Dobbs[1], M.A. Lawrence[1], A.J. Ramirez[1,2], M. Summerhayes[1], M.A. Richards[1], K.E. Towlson[1] & R.D. Rubens[1]

[1]*Imperial Cancer Research Fund Clinical Oncology Unit, Division of Oncology, and* [2]*Division of Psychiatry, United Medical and Dental Schools, Guy's Hospital, St Thomas Street, London SE1 9RT, UK.*

**Summary** Iododoxorubicin 80 mg $m^{-2}$ i.v. was given 3 weekly for a maximum of six cycles as first-line chemotherapy to 14 evaluable women with metastatic breast cancer. The response rate was 14% (95% confidence intervals 4-40%); median time to progression was 3.5 months (range 0.7 to >9.3) and median survival was 10.2 months (range 2.3 to >20.4). Neutropenia was the main toxicity but was not associated with severe sepsis. Two patients had a significant (>10%) but asymptomatic fall in cardiac ejection fraction; other toxicities were mild. Plasma pharmacokinetics was studied during the first cycle of treatment. Iododoxorubicin was extensively metabolised to iododoxorubicinol. Neutropenia and thrombocytopenia were both significantly correlated with the area under the concentration-time curve (AUC) for iododoxorubicin and the total AUC for iododoxorubicin and iododoxorubicinol. Quality of life (QOL), evaluated by self-report questionnaire and interview, showed little evidence of benefit in terms of physical symptom relief, level of activity, psychological symptoms or global evaluation of QOL during treatment. Iododoxorubicin is subjectively less toxic than standard anthracyclines, but at the dose and schedule used has limited activity in metastatic breast cancer, possibly because iododoxorubicinol is not clinically active.

Doxorubicin is one of the most important agents in the treatment of solid tumours. There has been considerable interest in the development of new anthracyclines, of which epirubicin and idarubicin have entered clinical use. There remains, however, a need for anthracyclines with either greater activity or reduced toxicity compared with those currently in use (Mross, 1991).

Iododoxorubicin (4′-iodo-4′-deoxydoxorubicin) differs from doxorubicin by the substitution of an iodine atom for the 4′-hydroxyl group on the daunosamine sugar. This results in iododoxorubicin having a lower $pK_a$ and increased lipophilicity under physiological conditions compared with doxorubicin (Barbieri *et al.*, 1987). Iododoxorubicin had greater activity than doxorubicin *in vitro* (Schwartz & Salmon, 1987) and was cytotoxic against cell lines resistant to doxorubicin (Barbieri *et al.*, 1987; Supino *et al.*, 1988; Schott *et al.*, 1990). *In vivo*, iododoxorubicin was more active than doxorubicin against the Lewis lung carcinoma (Barbieri *et al.*, 1987) and less cardiotoxic (Barbieri *et al.*, 1987; Danesi *et al.*, 1990). In phase I clinical trials (Gianni *et al.*, 1990; Mross *et al.*, 1990) severe neutropenia was the dose-limiting toxicity.

The phase II evaluation of a new cytotoxic drug typically concentrates on its clinical activity and toxicity. Recently, the importance of pharmacological studies in phase II clinical trials has been emphasised by Judson (1990). This may be the only opportunity to study the pharmacology of new drugs given as single agents. Those without activity will not be developed further and potentially useful cytotoxics may be discarded. Another important factor, especially in evaluating drugs to be used with palliative intent, is that conventional toxicity measures give an incomplete picture of the tolerability and acceptability of treatment to patients. This is best achieved by formal assessments of quality of life (Osoba, 1991).

This phase II study aimed to appraise fully the clinical activity, toxicity, pharmacology, and effect on quality of life of iododoxorubicin in patients with advanced breast cancer.

Correspondence: C. Twelves.
Received 14 May 1993; and in revised form 29 October 1993.

### Patients and methods

### *Patients*

Eligible women had histologically confirmed breast cancer with measurable metastatic or locally recurrent disease. They had not received prior chemotherapy for advanced disease but previous adjuvant chemotherapy excluding anthracyclines was permitted. Prior endocrine treatment and radiotherapy were allowed, provided no more than 30% of haemopoietically active marrow had been irradiated (Ellis, 1961). Other eligibility criteria included: WHO performance status 0-2, age 18-75 years, neutrophils $>2.0 \times 10^9 l^{-1}$ and platelets $>150 \times 10^9 l^{-1}$, and bilirubin $<35 \mu mol l^{-1}$ with serum transaminases no more than twice the upper limit of the reference range. Patients with a history of significant cardiac disease or a left ventricular ejection fraction (LVEF) below the normal range, brain metastases or a previous history of other malignancy were excluded.

Treatment toxicity was assessed according to WHO criteria (WHO, 1979) and response by UICC criteria (Hayward *et al.*, 1981). Response duration, progression-free interval and survival were measured from the date of the first cycle of iododoxorubicin.

All patients gave informed, written consent separately for the clinical study and pharmacokinetics. Both studies were approved by the Guy's Hospital ethics committee.

### *Treatment plan*

Patients received iododoxorubicin as an intravenous (i.v.) injection every 3 weeks, initially at a dose of 80 mg $m^{-2}$ over 2-5 min. Three patients, all of whom were more than 150% of their ideal body weight, received the first cycle of treatment at a dose based on their ideal surface area. Treatment was delayed if the blood count had not recovered. The dose was reduced by 10% in the event of clinically significant infection, WHO grade 3 or 4 mucositis or febrile neutropenia. Nadir blood counts were taken but dose adjustments were not made according to these counts alone. Patients routinely received metoclopramide 10 mg i.v. alone as antiemetic cover with other antiemetics given as necessary.

Prophylactic oral antibiotics were given at the discretion of the clinician during periods of neutropenia.

A maximum of six cycles of iododoxorubicin were given. Cardiac function was assessed clinically every 3 weeks. Gated cardiac (MUGA) scans were performed before treatment, after three cycles of chemotherapy and before each subsequent cycle. In patients developing congestive cardiac failure or a fall of more than 10% in LVEF iododoxorubicin was stopped.

### *Clinical pharmacology*

Thirteen blood samples were taken from each patient through an indwelling venous cannula over the 48 h following the first injection of iododoxorubicin, centrifuged and the plasma stored at $-20°C$.

Plasma levels of iododoxorubicin and its metabolites (iododoxorubicinol, 13-dihydroadriamycinone, 7-deoxyadriamycinone and 7-deoxy, 13-hydroadriamycinone) were measured by high-performance liquid chromatography (HPLC) and an advanced automated sample processor (AASP) using a method adapted from one used for epirubicin (Dobbs & Twelves, 1991). In brief, iododoxorubicin and its metabolites were extracted from 1 ml plasma samples onto prepared AASP $C_2$-cartridges which were introduced via the AASP into the stream of mobile phase [0.019 M sodium dihydrogen phosphate, pH 3.0, and acetonitrile at 1.86:1 (v/v), flow rate 0.8 ml $min^{-1}$]. Separation was achieved with a Lichrosorb RP-18 precolumn (particle size 5 $\mu m$, column dimensions 5 cm × 5 mm) and an Apex II ODS analytical column (particle size 2 $\mu m$, column dimensions 10 cm × 4.6 mm). Fluorescence detection was used (excitation and emission wavelengths 480 and 550 nm respectively); idarubicin was the internal standard.

The mean recovery of iododoxorubicin was 65%, and of its metabolites 71–94%. The routine detection limit was 5 ng $ml^{-1}$ for iododoxorubicin, and for the metabolites 1–5 ng $ml^{-1}$. The within-day and day-to-day precision of the assay were confirmed by coefficients of variation of less than 8% for iododoxorubicin and metabolites over a wide range of concentrations.

Iododoxorubicin pharmacokinetics was fitted to a three-compartment or a two-compartment model. The 'Pharmkit' programme (Johnson & Woollard, 1983) was used to obtain the early ($\alpha$), intermediate ($\beta$) and terminal ($\Gamma$) half-lives. Mean retention time (MRT, a measure of the period a molecule remains in the body) was calculated using 'Pharmkit'. The area under the concentration–time curve to 48 h ($AUC_t$) was calculated using the slopes and intercepts derived from 'Pharmkit', extrapolated back to the end of the injection and corrected for the duration of the injection (Freedman & Workman, 1988). Elimination of iododoxorubicin from the plasma was expressed as drug clearance ($Cl = dose/AUC_L$). The volume of distribution of iododoxorubicin was calculated as $V_{ss} = dose \times AUMC/AUC^2$ (Benet & Galeazzi, 1979), where AUMC is the area under the first moment curve.

The $AUC_t$ of the metabolites were measured; the ratio, $R$, of the $AUC_t$ of the metabolites to the $AUC_t$ of iododoxorubicin was calculated.

### *Quality of life*

Assessment of quality of life involved the patient's report of physical and psychological symptoms, levels of physical activity, a global evaluation of quality of life and practical difficulties associated with treatment. These were assessed using a self-report questionnaire, the Rotterdam Symptom Check List (RSCL; de Haes *et al.*, 1990) administered before treatment and 6 weeks after the first cycle of chemotherapy. A semistructured interview was also conducted after the completion of treatment.

The RSCL includes these seven items concerned with psychological symptoms: feeling worried, irritable, nervous, depressed, anxious, tense and despondent about the future. It also incorporates a range of physical symptoms and a global rating of performance status. Patients were asked to rate these items according to their experience during the previous week. The severity of common side-effects of chemotherapy in the days after treatment was also elicited. Each item is scored between 0 (not at all) and 3 (very much). An interview after the treatment course had been completed enquired about problems arising from practical aspects of treatment. Patients were also asked how worthwhile they felt their treatment had been and whether overall they felt better, the same or worse than just before starting chemotherapy.

### *Statistical methods*

The protocol specified a response rate of 60% for demonstrating significant clinical activity. Time to progession and survival were calculated from the start of chemotherapy (Kaplan & Meier, 1958).

Pharmacokinetic parameters were correlated with haematological parameters measured following the first course of treatment. Those pharmacokinetic parameters which were not normally distributed were expressed on a $\log_{10}$ scale. The surviving fraction (SF) of blood cells was expressed on a $\log_{10}$ scale since this is linearly related to drug exposure *in vitro* (Skipper *et al.*, 1970). Pearson's correlation was used to measure the extent of the pharmacodynamic relationships. A correlation coefficient ($r$) $>0.5$ was considered to show significant predictive capability since under those circumstances 25% of the variability in the clinical value could be accounted for by changes in the pharmacokinetic parameter.

Changes in quality of life scores between the pretreatment and 6 week assessment were analysed using the Wilcoxon signed-rank test. The concordance between the patient's and clinician's reports of alopecia and nausea and vomiting was assessed using Fisher's exact test.

### Results

### *Clinical activity and toxicity*

Sixteen woman were treated between April and December 1991. Two patients, one who declined to attend for 8 weeks following her first cycle of iododoxorubicin and one later found to have a past history of ovarian cancer, were excluded from the analyses. The pretreatment characteristics of the remaining 14 evaluable women are shown in Table I.

These 14 patients all received at least two cycles of treatment. Four completed the planned six cycles of chemotherapy and seven discontinued treatment because of progressive disease. The three remaining patients, who had stable disease, stopped iododoxorubicin because of unacceptable toxicity (two WHO grade 3 or 4 vomiting, one $>15\%$ fall in LVEF). A total of 55 cycles of iododoxorubicin were administered, of which ten cycles were either delayed or given at a reduced dose. Neutropenia accounted for seven of the delays or dose reductions, anaemia for two, infection and/or malaise for two and anticipatory vomiting for one delay.

The median follow-up period was 12.0 months. No patient had a complete response, but two achieved a partial response and a further eight had stable disease. The response rate was, therefore, 2/14 (14%, with 95% confidence intervals 4–40%); response durations were 4.5 and $>5.6$ months. In all, four responses were seen (three in lymph nodes, one pulmonary metastasis) from a total of 25 measurable disease sites (16%). The median time to progression for all 14 evaluable patients was 3.5 months (range 0.7–11.3 months); median survival was 10.2 months (range 2.3 to $>20.4$ months).

The worst toxicity experienced by each patient is shown in Table II. Neutropenia was the main toxicity and WHO grade 3 or 4 toxicity was associated with 35/55 (64%) treatment cycles. There were 16 episodes of infection in nine patients, 11 of which were associated with a neutrophil count less than

**Table I** Characteristics of evaluable patients ($n = 14$)

| *Median age* | *59 (range 39–66)* |
|---|---|
| ECOG performance status: | |
| 0 | 3 |
| 1 | 11 |
| Histology | |
| Infiltrating ductal | 9 |
| Infiltrating lobular | 1 |
| Other/unknown | 4 |
| Receptor status: | |
| ER positive | 5 |
| negative | 4 |
| unknown | 5 |
| PR positive | 2 |
| negative | 7 |
| unknown | 5 |
| Prior systemic treatment | |
| Adjuvant endocrine | 6 |
| Adjuvant chemotherapy | 2 |
| Advanced endocrine | 10 |
| Percentage active marrow previously irradiated | |
| None | 3 |
| 1–10 | 8 |
| >10 | 3 |
| Measurable disease sites[a] | |
| Cutaneous | 4 |
| Lympathic | 6 |
| Breast | 5 |
| Bone | 6 |
| Visceral | 3 (two lung), one (liver) |
| Other | 1 (pleural) |

[a]Nine patients had more than one site of measurable disease. There were a further six evaluable, but not measurable, sites of disease.

**Table II** Worst toxicity experienced (out of 14 patients)

| | *WHO grade* | | | | |
|---|---|---|---|---|---|
| *Toxicity* | *0* | *1* | *2* | *3* | *4* |
| Neutropenia | 0 | 0 | 1 | 5 | 8 |
| Thrombocytopenia | 6 | 2 | 4 | 2 | 0 |
| Anaemia | 0 | 7 | 5 | 2 | 0 |
| Infection | 5 | 8 | 1 | 0 | 0 |
| Stomatitis | 7 | 5 | 2 | 0 | 0 |
| Nausea/vomiting | 2 | 1 | 7 | 2 | 2 |
| Alopecia | 4 | 9 | 1 | 0 | 0 |

$1.0 \times 10^9 l^{-1}$, but only one patient was admitted to hospital whilst neutropenic. Four patients required oral antibiotics for infections but none received intravenous antibiotics.

Although most patients received metoclopramide as the only antiemetic, the majority of cycles of chemotherapy were not associated with clinically significant nausea and vomiting. Intractable (WHO grade 4) vomiting was, however, recorded in two patients, one of whom had experienced severe emesis with adjuvant chemotherapy and later required psychiatric intervention. The median change in LVEF during treatment was − 1.5% (range + 18.3% to − 24.1%). Two women, one of whom had received radiotherapy to the left breast, had falls in LVEF of 15% and 24.1% after 160 and 320 mg $m^{-2}$ of iododoxorubicin respectively. Neither patient developed clinical evidence of cardiac failure or ECG abnormalities. Alopecia, judged by WHO criteria, was mild and stomatitis was uncommon. One patient experienced an extravasation causing pain, swelling and erythema which resolved without ulceration.

### *Pharmacokinetics and pharmacodynamics*

In ten women iododoxorubicin pharmacokinetics fitted a three-compartment model and the remaining four fitted a two-compartment model. A typical concentration–time curve from one patient is shown in Figure 1. The pharmacokinetics parameters for iododoxorubicin and its metabolites are shown in Table III. Iododoxorubicin clearance and $R$ for iododoxorubicinol ($R_{\text{Iododox-ol}}$) were significantly correlated with body weight ($r = 0.52$, $P = 0.03$, and $r = 0.65$, $P = 0.006$, respectively) but not with other morphometric or biochemical parameters.

Relationships between the $AUC_t$ of iododoxorubicin and iododoxorubicinol and nadir counts after the first cycle of treatment, represented as absolute values and surviving fractions (SF), are shown in Tables IVa and IVb respectively. The SF for neutrophils (Figure 2), total WBC and platelets all correlated with iododoxorubicin $AUC_t$, as did absolute nadir neutrophil aand platelet counts. In addition, the five patients with WHO grade 4 neutropenia after their first cycle of chemotherapy had a significantly higher median iododoxorubicin $AUC_t$ than the eight with lesser degrees of neutropenia (532 and 352 ng $ml^{-1}$ h respectively, $P = 0.01$). In a multivariate analysis only iododoxorubicin $AUC_t$ predicted for neutropenia ($r^2 = 44.6\%$). Body weight, height, surface area, pretreatment blood counts, age, and extent of prior radiotherapy did not predict for neutropenia.

Neither peak iododoxorubicin plasma concentration nor $AUC_t$ correlated with the severity of nausea and vomiting during the first cycle of treatment or the subsequent fall in LVEF. Only two patients responded to iododoxorubicin, so it was not possible to correlate response to treatment with pharmacokinetics.

### *Quality of life*

All 14 evaluable patients underwent initial quality of life assessment; 12 were also evaluable at 6 weeks (two patients who had discontinued iododoxorubicin did not attend). Eleven patients later had a post-treatment interview.

The median RSCL psychological score for all 14 patients prior to treatment was 10 (range 0–17). There was no significant change in the median RSCL psychological score for the 12 patients who completed both the pretreatment and 6 week assessments ($P = 0.13$). Levels of activity prior to treatment and at 6 weeks appeared to be unchanged (12/14 and 11/12 respectively 'up all day'; 2/14 and 0/12 respectively 'up half the day'; 0/14 and 1/12 respectively 'up only for short periods' or 'confined to bed').

The most commonly reported physical symptoms and their prevalence in the week prior to the pretreatment and 6 week assessments are shown in Table V. Tiredness and lack of energy were the most prevalent symptoms, with more than half of patients complaining of the former and about a third complaining of the latter at both time points. In addition, 5/12 (42%) patients described moderate or severe nausea, while 1/12 (17%) described similar levels of vomiting in the days after the second cycle of treatment. There was no significant difference in the frequency of alopecia or nausea

**Figure 1** Plasma profile of iododoxorubicin and metabolites in a patient following 80 mg $m^{-2}$ iododoxorubicin. (iododoxorubicin, ————; iododoxorubicinol, – – – –; 13-dihydroadriamycinone, ——).

**Table III** Pharmacokinetic parameters for iododoxorubicin and its metabolites

| Parameter | I-DOX | I-DOXOL | 7-A[a] | 13-A | 7, 13-A[b] |
|---|---|---|---|---|---|
| | | | *Mean value (s.d.)* | | |
| $AUC_t$ ($ng\ ml^{-1}\ h$) | 478.3 (315) | 4294.0 (1227) | 68.0 (69) | 363 (439) | 135 (89) |
| PPC ($ng\ ml^{-1}$) | 481.5 (188) | 512.2 (216) | 11.6 (9) | 22.4 (13) | 14 (12) |
| TPPC (min) | 7.8 (2.6) | 11.6 (7.7) | 4.0 (8) | 24.4 (60) | 120 (150) |
| MRT (h) | 20.7 (15) | – | – | – | – |
| Clearance ($l\ h^{-1}$) | 319.0 (142) | – | – | – | – |
| Vss (l) | 5479.0 (3504) | – | – | – | – |
| $a - t_{1/2}$ (h) | 0.16 (0.17) | – | – | – | – |
| $b - t_{1/2}$[c] (h) | 0.80 (0.3) | – | – | – | – |
| $c - t_{1/2}$ (h) | 20.6 (12.0) | 11.6 (2.5) | 24.2 (18) | 20.6 (18) | 36 (48) |
| R | – | 11.8 (3.8) | 0.2 (0.3) | 0.9 (1.0) | 0.4 (0.3) |

Abbreviations: I-DOX, iodoxodorubicin; I-DOXOL, iodoxodorubicin; 7-A, 7-deoxyadriamycinone; 13-A, 13-dihydoadriamycinone; 7, 13-A, 7-deoxy, 13-dihydroadriamycinone.
[a]$n = 8$ patients; 7-A not detected in six women. [b]$n = 13$ patients; 7, 13-A not detected in one woman. [c]$b - t_{1/2}$ from 10 patients fitted to a three-compartment model.

**Table IVa** Relationship between absolute nadir blood counts and AUCs ($n = 14$)

| AUC | r (*P-value*) *Haemoglobin* | *Total WBC* | *Neutrophils* | *Platelets* |
|---|---|---|---|---|
| | | *Correlation coefficients* | | |
| I-DOX | – 0.07 (0.41) | – 0.40 (0.07) | **– 0.54 (0.023)** | **– 0.60 (0.01)** |
| I-DOXOL | – 0.16 (0.29) | – 0.38 (0.09) | **– 0.58 (0.014)** | – 0.43 (0.06) |
| I-DOX + I-DOXOL | – 0.15 (0.373) | – 0.41 (0.07) | **– 0.62 (0.01)** | **– 0.50 (0.03)** |

**Table IVb** Relationship between the surviving fraction (SF) nadir blood counts and AUCs ($n = 14$)

| AUC | r (*P-value*) *Haemoglobin* | *Total WBC* | *Neutrophils* | *Platelets* |
|---|---|---|---|---|
| | | *Correlation coefficients* | | |
| I-DOX | 0.29 (0.16) | **– 0.65 (0.001)** | **– 0.67 (0.004)** | **– 0.73 (0.002)** |
| I-DOXOL | 0.42 (0.07) | – 0.33 (0.13) | – 0.45 (0.05) | **– 0.59 (0.012)** |
| I-DOX + I-DOXOL | 0.40 (0.08) | – 0.43 (0.06) | **– 0.53 (0.026)** | **– 0.66 (0.005)** |

Values in bold are significant as defined in the text.

**Figure 2** Relationship between $\log_{10}$ $AUC_t$ for iododoxorubicin and $\log_{10}$ SF neutrophils.

and vomiting (both $P = 0.2$) as reported by the patient and the clinician. Two of 12 (17%) reported moderate or severe hair loss at the time. However, one patient described her hair loss as severe, while the clinician rated it as only WHO grade 1.

The most commonly reported difficulties arising from practical aspects of treatment were related to the investigations; half the patients reported moderate or severe problems. The

**Table V** Numbers of patients scoring moderately or highly on key physical items of RSCL

| *RSCL question* | *Pretreatment* n = *14 (%)* | *Six weeks* n = *12 (%)* |
|---|---|---|
| Been tired? | 7 (50) | 7 (58) |
| Been lacking energy? | 4 (29) | 4 (33) |
| Been short of breath? | 2 (14) | 4 (33) |
| Been in pain? | 6 (43) | 2 (17) |
| Been lacking appetite? | 3 (21) | 0 (0) |
| Had a dry mouth? | 3 (21) | 0 (0) |

tests were described as time-consuming and the MUGA scan as particularly unpleasant. Five of the 11 patients described significant problems with the chemotherapy injection iself, including difficulties with venepuncture and an extravasation. In three women these problems occurred following cannulation by an inexperienced member of the medical staff; subsequent cannulations by a nurse specialist were described as less problematic. Other problems related to frequent hospital visits (three patients), arranging transport (one), parking (five) and taking time off work (two).

After completing chemotherapy, four patients felt the same, seven felt worse and none felt better than they did before treatment. At this time eight patients felt chemotherapy had not been at all worthwhile, one a little worthwhile, one moderately worthwhile and only one judged it as very worthwhile. Neither of the patients who responded felt treatment had been at all worthwhile.

### Discussion

This study, comprising clinical, pharmacokinetic and quality of life parameters, is a comprehensive evaluation of iododoxorubicin in patients with advanced breast cancer. The study closed after 14 evaluable patients had been treated following a scheduled analysis at which the response rate was 14% (95% CI 4-40%). Although the confidence intervals are wide, this response rate is much lower than the 60% response rate defined in the protocol as representing significant activity for iododoxorubicin in advanced breast cancer in comparison with other anthracyclines. Nevertheless, the study provided useful pharmacological and quality of life data.

The 14% response rate is disappointing given the preclinical activity of iododoxorubicin (Barbieri *et al.*, 1987; Schwartz & Salmon, 1987; Supino *et al.*, 1988; Schott *et al.*, 1990). In the earlier phase II studies, Sessa *et al.* (1991) reported a response rate of 2/20 (10%) in women with metastatic breast cancer. This is lower than that reported by Gianni *et al.* (1991); the response rate was 11/31 (35%) in women with locally advanced disease and 5/15 (33%) in women with metastatic disease. Despite the higher response rate, the median response duration was only 53 days in the latter study (Gianni *et al.*, 1991). Schwartsmann and Pinedo (1991) suggested that problems of patient selection and dose intensity in the multicentre study (Sessa *et al.*, 1991) may explain the lower response rate compared with the single-centre study from Milan (Gianni *et al.*, 1991).

The current study helps to clarify the activity of iododoxorubicin in metastatic breast cancer. Iododoxorubicin dose intensity and the frequency of severe neutropenia were very similar to that seen by the Milan group. The substantially lower response rate in the current study is, therefore, probably the result of patient selection. In the 49 women with metastatic breast cancer reported in the three phase II studies, a total of nine partial responses have been seen (18%, CI 10-31%). Together these studies indicate that iododoxorubicin given 3 weekly at these doses is less active than either doxorubicin or epirubicin given at standard doses as first-line chemotherapy for metastatic breast cancer.

The pharmacological data obtained in this phase II study broadly confirm those of the phase I studies (Gianni *et al.*, 1990; Mross *et al.*, 1990; Robert *et al.*, 1992). Iododoxorubicin was extensively metabolised to iododoxorubicinol with the aglycone metabolites being less prominent. The correlation between $R_{\mathrm{Iododox\text{-}ol}}$ and weight has not been described previously. Metabolic clearance due to conjugation appears to be enhanced in the obese (Abernathy *et al.*, 1982). The correlation between both $R_{\mathrm{Iododox\text{-}ol}}$ and clearance of iododoxorubicin with body weight may be due to increased activity of the widely distributed aldo-keto reductases, which metabolise anthracyclines to their alcohols (Loveless *et al.*, 1978). If iododoxorubicinol is less 'active' than the parent compound in man, obese patients may be undertreated if dosage is based on their ideal, or even actual, surface area.

The high value of $R_{\mathrm{Iododox\text{-}ol}}$ emphasises the importance of iododoxorubicinol in determining the activity in patients treated with iododocorubicin. Although *in vitro* the cytotoxicity of iododoxorubicinol is equal to that of iododoxorubicin (Schott *et al.*, 1990), Robert *et al.*, (1992) showed that iododoxorubicinol pentrates human tumours *in vivo* much less than iododoxorubicin. The relatively low response rates to iododoxorubicin in advanced breast cancer suggest that, although the parent compound may be cytotoxic, metabolism to iododoxorubucinol reduces its clinical activity. Interestingly, in mice there is much less metabolism of iododoxorubicin to iododoxorubicinol (Formelli *et al.*, 1987), and interestingly iododoxorubicin is active against murine tumours *in vivo* (Barbieri *et al.*, 1987).

Given the disappointing response rate, it is not surprising there is little evidence that benefit accrued from treatment in terms of quality of life. In line with the clinical evaluation, there was little evidence of change in common physical symptoms during the first 6 weeks of treatment with the possible exception of an improvement in pain. Similarly, activity levels and levels of psychological distress remained largely unchanged during that time. Three-quarters of the patients reported that they felt treatment was only 'a little' or 'not at all' worthwhile. None of the patients felt better than before starting treatment, and over half felt worse. This study demonstrated how qualify of life data can be collected in a phase II study, but the value of such data would be better assessed where the agent under evaluation had activity which had to be balanced against toxicity. Under those circumstances QOL data may complement the routinely collected clinical toxicity data.

Enhanced activity is not the only goal for new anthracyclines, and altered patterns of toxicity may also be important. With the exception of myelosuppression, WHO treatment-related toxicities and patients' reported side-effects were generally mild. Stomatitis, alopecia, nausea and vomiting were much less severe than with established anthracyclines given at maximum tolerated doses. The generally good agreement between the patient's and doctor's assessment of alopecia and nausea and vomiting is encouraging. However, patients found that practical aspects of the study caused difficulties. The burden on patients of the additional investigations, which are a frequent requirement of phase II trials, should be considered in the design of these studies.

The dose-limiting toxicity of iododoxorubicin, myelosuppression, is clearly related to $AUC_t$. By contrast with the phase I data (Robert *et al.*, 1992), myelosuppression was more strongly correlated with the AUC of iododoxorubicin than with that of iododoxorubicinol. Ackland *et al.* (1989) reported a correlation between neutropenia and doxorubicin pharmacokinetics, but such relationships have not been widely demonstrated. The fall in LVEF seen in two patients during the current study at relatively low cumulative doses of iododoxorubicin was unexpected. Only 2 of 96 patients in the earlier phase II studies had similar changes (Gianni *et al.*, 1991; Sessa *et al.*, 1991), and in animal models iododoxorubicin is substantially less cardiotoxic than doxorubicin (Danesi *et al.*, 1990). Although neither patient developed symptoms of heart failure, cardiac function should be monitored carefully in future studies.

Taken together, the three published studies suggest modest activity for iododoxorubicin 80 mg $ml^{-2}$ in comparison with equitoxic doses of doxorubicin or epirubicin. Although iododoxorubicin has fewer subjective side-effects than other anthracyclines, at this dose and schedule it appears to be significantly less active than doxorubicin and epirubicin against metastatic breast cancer. This was confirmed by the failure of psychological distress, physical symptoms and overall quality of life to improve following treatment. This low response rate may be due to the extensive metabolism of iododoxorubicin to iododoxorubicinol, which may not be clinically active. A role for iododoxorubicin in the treatment of patients with advanced breast cancer remains to be identified. It may be possible to give iododoxorubicin at higher doses, especially with the use of colony-stimulating factors, but such regimens are unlikely to be more cost-effective than conventional anthracyclines.

The current study shows how a comprehensive assessment of a new agent can be achieved by combining evaluation of clinical activity, pharmacokinetics and quality of life in a single-centre phase II trial. We commend this as a model for the evaluation of new cytotoxic agents.

We are grateful to Dr W.M. Gregory for assistance with the statistical analyses. Iododoxorubicin and pure analytic standards were provided by Farmitalia Carlo Erba (Milan, Italy). N.A. Dobbs and M.A. Lawrence are supported in part by a grant from Farmitalia Carlo Erba.

### References

ABERNATHY, D.R., DIVOLI, M., NARMATZ, J.S. & SHADES, R.I. (1982). Obesity, sex and acetominophen disposition. *Clin. Pharmacol. Ther.*, **31**, 783.

ACKLAND, S.P., RATAIN, M.J., VOZELGANG, N.J., CHOI, K.E., RUANE, M. & SINKULE, J.A. (1989). Pharmacokinetics and pharmacodynamics of long-term infusion doxorubicin. *Clin. Pharmacol. Ther.*, **45**, 340–347.

BARBIERI, B., GIULIANI, F.C., BORDONI, T., CASAZZA, A.M., GERONI, C., BELLINI, O., SUARATO, A., GIOIA, B., PENCO, S. & ARCAMONE, F. (1987). Chemical and biological characterization of 4′-iodo-4′-deoxydoxurubicin. *Cancer Res.*, **47**, 4001–4006.

BENET, L.Z. & GALEAZZI, R.L. (1979). Noncompartmental determination of the steady-state volume of distribution. *J. Pharmacol. Sci.*, **68**, 1071.

DANESI, R., MARCHETTI, A., BERNARDINI, N., LA ROCCA, R.V., BEVILACQUA, G. & DEL TACCA. M. (1990). Cardiac toxicity and antitumor activity of 4′-deoxy-4′-iodo-doxorubicinol. *Cancer Chemother. Pharmacol.*, **26**, 403–408.

DE HAES, J., VAN KNIPPENBERG, F. & NEIJT, J. (1990). Measuring psychological and physical distress in cancer patients: structure and application of the Rotterdam Symptom Check List. *Br. Med. J.*, **62**, 1034–1038.

DOBBS, N.A. & TWELVES, C.J. (1991). Measurement of epirubicin and its metabolites by high-performance liquid chromatography using an advanced sample processor. *J. Chrom. Biomed. Appl.*, **572**, 37–42.

ELLIS, R.E. (1961). The distribution of active bone marrow in the adult. *Phys. Med. Biol.*, **5**, 255–258.

FORMELLI, F., CARSANA, R. & POLLINI, C. (1987). Pharmacokinetics of 4′-deoxy-4′-iodo-doxorubicin in plasma and tissues of tumor-bearing mice compared with doxorubicin. *Cancer Res.*, **47**, 5401–5406.

FREEDMAN, L.S. & WORKMAN, P. (1988). When can the infusion period be safely ignored in the estimation of pharmacokinetic parameters of drugs in humans? *Cancer Chemother. Pharmacol.*, **22**, 95–103.

GIANNI, L., VIAGANO, L., SURBONE, A., BALLINARI, D., CASALI, P., TARELLE, C., COLLINS, J.M. & BONADONNA, G. (1990). Pharmacology and clinical toxicity of 4′-iodo-4′-deoxydoxorubicin: an example of successful application of pharmacokinetics to dose escalation in phase I trials. *J. Natl. Cancer Inst.*, **82**, 469–477.

GIANNA, L., CAPRI, G., GRECO, M., VILLANI, F., BRAMBILLA, C., LUINI, A., CRIPPA, F. & BONADONNA, G. (1991). Activity and toxicity of 4′-iodo-4′-deoxydoxorubicin in patients with advanced breast cancer. *Ann. Oncol.*, **2**, 719–725.

HAYWARD, J.L., CARBONE, P., HEUSON, J.C., KUMAOKA, S., SEGALOFF, A. & RUBENS, R.D. (1981). Assessment of response in advanced breast cancer. *Eur. J. Cancer*, **13**, 89–94.

JOHNSON, A. & WOOLLARD, R.C. (1983). Stripe: an interactive computer programme for the analysis of drug pharmacokinetics. *J. Pharmacol. Methods*, **9**, 193–200.

JUDSON, I.R. (1990). Phase II studies: wrong doses, wrong patients? *Eur. J. Cancer*, **27**, 1198–1200.

KAPLAN, E.L. & MEIER, P. (1958). Nonparametric estimation from incomplete observations. *J. Am. Stat. Assoc.*, **53**, 457–481.

LOVELESS, R., ARENA, E., FELSTED, R.L. & BACHUR, N.R. (1978). Comparative mammalian metabolism of adriamycin and duanorubicin. *Cancer Res.*, **38**, 593–598.

MROSS, K. (1991). New anthracycline derivatives: what for? *Eur. J. Cancer*, **27**, 1542–1544.

MROSS, K., MAYER, U., LANGENBUCH, T., HAMM, K., BURK, K. & HOSFIELD, D. (1990). Toxicity, pharmacokinetics and metabolism of iododoxorubicin in cancer patients. *Eur. J. Cancer*, **26**, 1156–1162.

OSOBA, A. (ed.) (1991). *Effect of Cancer on Quality of Life*. CRC Press: Boca Raton, FL.

ROBERT, J., ARMAND, J.P., HUET, S., KILINK-ALAKI, M., RECONDO, G. & URTELOUP, P. (1992). Pharmacokinetics and metabolism of 4′-iodo-4′-deoxy-doxorubicin in humans. *J. Clin. Oncol.*, **10**, 1183–1190.

SCHOTT, B., VRIGNAUD, P., RIES, C., ROBERT, J. & LONDOS-GALIARDI, D. (1990). Cellular pharmacology of 4′-iodo-4′-deoxydoxorubicin. *Br. J. Cancer*, **61**, 543–547.

SCHWARTSMANN, G. & PINEDO, H.M. (1987). Clinical trials in advanced breast cancer. *Eur. J. Cancer Clin. Oncol.*, **23**, 595–597.

SCHWARTSMANN, G. & PINEDO, H.M. (1991). Multicentre vs single centre phase II evaluation of 4′-iodo-4′-deoxydoxorubicin in advanced breast cancer. *Ann. Oncol.*, **2**, 703–706.

SCHWARTZ, J.E. & SALMON, S.E. (1987). Comparative *in vitro* activity of 4′-deoxy-4′-iodo-doxorubicin and other anthracyclines in the human tumor clonogenic assay. *Invest. New Drugs*, **5**, 231–234.

SESSA, C., CALABRESI, F., CAVALLI, F., CERNY, T., LIATI, P., SKOVSGAARD, T., SORIO, R. & KAYE, S.B. (1991). Phase II studies of 4′-iodo-4′-deoxydoxorubicin in advanced non-small cell lung, colon and breast cancers. *Ann. Oncol.*, **2**, 727–731.

SKIPPER, H.E., SCHABEL, F.M. & MELLETT, L.B. (1970). Implications of biochemical, cytokinetic, pharmacologic, and toxicologic relationships in the design of optimal therapeutic schedules. *Cancer Chemother. Rep.*, **54**, 431–450.

SUPINO, R., MARIANI, M., PROSPERI, E. & PARMIANI, G. (1988). Lack of cross-resistance of a doxorubicin-resistant B16 melanoma cell line with 4′-deoxy-4′-iodo-doxorubicin. *Cancer Chemother. Pharmacol.*, **21**, 251–254.

WHO (1979). *WHO Handbook for Reporting Results of Cancer Treatment*. World Health Organization: Geneva.